# European propolis is highly active against trypanosomatids including *Crithidia fasciculata*

**DOI:** 10.1038/s41598-019-47840-y

**Published:** 2019-08-06

**Authors:** Abdullah Alotaibi, Godwin U. Ebiloma, Roderick Williams, Samya Alenezi, Anne-Marie Donachie, Selome Guillaume, John O. Igoli, James Fearnley, Harry P. de Koning, David G. Watson

**Affiliations:** 1 University of Strathclyde, Strathclyde Institute of Pharmacy and Biomedical Science, 161 Cathedral Street, Glasgow, G4 0RE UK; 2 Institute of Infection, Immunity and Inflammation, College of Medical, Veterinary and Life Sciences, University of Glasgow, Glasgow, G12 8TA UK; 3 IBEHR, School of Health and Life Science, University of the West of Scotland, High Street, Paisley, PA1 2BE UK; 4 Department of Chemistry, University of Agriculture, PMB 2373 Makurdi, Nigeria; 5 BeeVital, Whitby, North Yorkshire YO22 5JR UK

**Keywords:** Mass spectrometry, Antiparasitic agents

## Abstract

Extracts of 35 samples of European propolis were tested against wild type and resistant strains of the protozoal pathogens *Trypanosoma brucei*, *Trypanosoma congolense* and *Leishmania mexicana*. The extracts were also tested against *Crithidia fasciculata* a close relative of *Crithidia mellificae*, a parasite of bees. *Crithidia*, *Trypanosoma* and *Leishmania* are all members of the order Kinetoplastida. High levels of activity were obtained for all the samples with the levels of activity varying across the sample set. The highest levels of activity were found against *L. mexicana*. The propolis samples were profiled by using liquid chromatography with high resolution mass spectrometry (LC-MS) and principal components analysis (PCA) of the data obtained indicated there was a wide variation in the composition of the propolis samples. Orthogonal partial least squares (OPLS) associated a butyrate ester of pinobanksin with high activity against *T. brucei* whereas in the case of *T. congolense* high activity was associated with methyl ethers of chrysin and pinobanksin. In the case of *C. fasciculata* highest activity was associated with methyl ethers of galangin and pinobanksin. OPLS modelling of the activities against *L. mexicana* using the mass spectrometry produced a less successful model suggesting a wider range of active components.

## Introduction

Propolis is a resinous substance collected by bees, generally from plant buds. Its composition varies widely according to the vegetation surrounding the bee hive^1^. It is collected on the hindlegs of the bee and is removed with the help of other bees upon return to the hive and layered onto surfaces and used to fill any gaps within the hive, helping to maintain a sterile environment within the hive. In Northern Europe and other temperate regions such as Northern China and North America propolis is generally collected from the buds of poplar species^1,2^, whereas in Southern Europe the predominant sources are various Cypress species and in tropical regions several different plant sources may be utilised^1,3^. Propolis almost always displays high activity against *Trypanosoma brucei* and other protozoa, particularly those from the order Kinetoplastida, and we have found this to be the case regardless of the region of origin. Antiprotozoal activity has been found in propolis from Libya, Nigeria, Cameroon, Saudi Arabia and Brazil^3–10^. Although propolis is also antibacterial this activity is often only moderate in most samples and absent in others; generally, the strongest antibacterial activity is found in tropical propolis samples^11,12^. It has recently become clear that protozoal infection in bees is widespread, this was originally thought to be caused by *Crithidia mellificae*, which has been found to be associated with a higher incidence of winter colony collapse in Belgian bee colonies^13^, but it is now thought that the protozoal species *Lotmaria passim*^14,15^ is the main infecting organism. It has been found that DNA from *L. passim* is the most abundant DNA from a pathogenic organism within the DNA profile for the microbiome of Scottish bees^16^. Recently, *L. passim* has also been found in Africanised bees from Argentina, Uruguay and Chile and in this report a heavy burden of infection was found to be associated with a higher incidence of Varroa mite infestation^17^. Thus far there is no evidence that bees ingest propolis but since the spread of the protozoal infection occurs via faeces, coating the surfaces in the hive with propolis that is active against trypanosomatids could prevent transmission^18^. It remains an unanswered question just how important propolis is to the bee, and what its exact mechanism is in keeping down infections within the hive. European propolis has been extensively characterised and is composed of a complex mixture of >300 flavonoids and cinnamic acid derivatives^19,20^ and even though it has been worked on for many years there still remain components in it that have not been completely chemically or biologically characterised^20^, especially with regards to their antimicrobial properties. In this paper we report the activity of 35 European propolis samples against *Trypanosoma brucei*, *Trypanosoma congolense*, *Leishmania mexicana* and *Crithidia fasciculata*.

## Results

**Figure 1:**
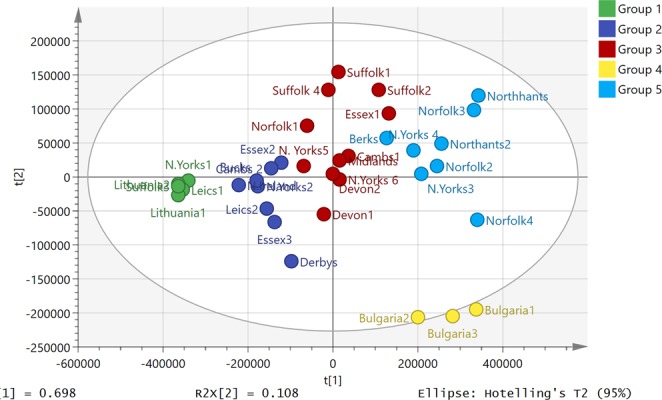
PCA plot showing the variation of propolis composition across 35 European propolis samples (Pareto scaled based on 233 components).

**Figure 2:**
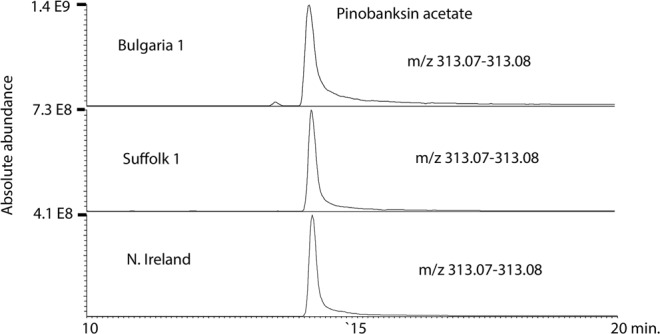
Extracted ion trace showing variation in the levels of pinobanksin acetate across 3 European propolis samples.

**Figure 3:**
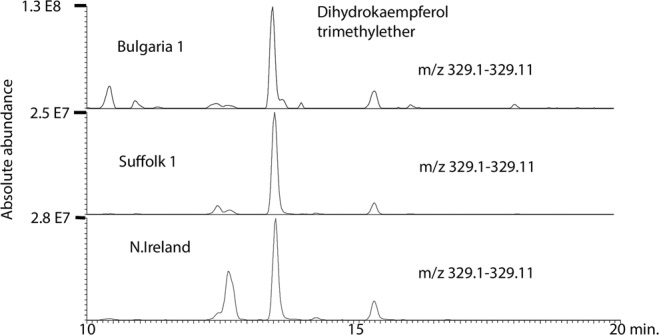
Extracted ion trace showing variation in trimethyl dihydrokaempferol across 3 European propolis samples.

Figure 1 shows the spread of the compositions of the propolis samples in a PCA model. We have previously characterised most of the major components in propolis from the UK by using accurate mass measurement with LC-MS^n^^20^. Although the samples have broadly similar compositions, there are some quite marked variations in individual components. For instance, Fig. 2 shows extracted ion chromatograms for a major component, pinobanksin acetate, across three samples from different positions in the PCA plot. Pinobanksin acetate is most abundant in the Bulgarian samples, which contain ~3.5-fold more of the compound than a sample from Northern Ireland. In contrast, Fig. 3 shows extracted ion traces for a component putatively identified as trimethyl dihydrokaempferol, which is abundant in the Northern Ireland sample but only present at low levels in the Bulgarian sample. Table 1 shows the results obtained in testing the 35 samples of European propolis against *Trypanosoma brucei, Trypanosoma congolense* and the multidrug resistant strain *Trypanosoma brucei B48*. Of these, 4 samples displayed high activity, i.e. EC_50_ values < 5 µg/mL, and 21 displayed intermediate activity between 5 and 10 µg/mL for the standard drug-sensitive strain Lister 427WT. The propolis samples from Norfolk displayed the highest activity, followed by the adjoining county of Suffolk and nearby Northamptonshire. The EC_50_ values for the multidrug resistant stain B48 were within ~1.5-fold of the control (Resistance Index (RI) 0.63–1.56; average 0.83 ± 0.04) although the RI for pentamidine was 222 (P < 0.001, Student’s unpaired t-test; Table 1).

**Table 1:** The activity (µg/ml) of 35 European propolis samples against the standard drug-sensitive *T. brucei* 427WT and multi-drug resistant strain *T. brucei* B48, and *T. congolense*.

Propolis sample	*T. brucei*				*T. congolense*
	427WT EC_50_	B48 EC_50_	R.I.	P value	IL3000 EC_50_
Suffolk 4, UK	7.42 ± 0.37	5.7 ± 0.17	0.77	0.013	8.46 ± 1.47
Bulgaria 1	5.20 ± 0.18	3.6 ± 0.52	0.69	0.043	3.69 ± 0.79
Suffolk 2, UK	6.69 ± 0.36	7.7 ± 1.1	1.15	0.423	5.66 ± 1.55
North Yorkshire 1, UK	13.5 ± 0.61	11.0 ± 0.70	0.82	0.058	18.9 ± 1.1
Northamptonshire 1, UK	4.49 ± 0.22	3.0 ± 0.20	0.67	0.007	5.69 ± 1.10
Essex 1, UK	5.97 ± 0.17	4.6 ± 0.26	0.77	0.013	4.40 ± 0.47
Essex 2, UK	14.0 ± 0.13	10.6 ± 1.6	0.75	0.102	17.3 ± 2.4
Norfolk 1, UK	5.23 ± 0.49	3.3 ± 0.31	0.63	0.029	3.08 ± 0.90
Devon 1, UK	8.57 ± 0.26	10.8 ± 1.2	1.26	0.144	11.4 ± 1.8
Leicestershire 1, UK	13.7 ± 1.18	11.6 ± 2.3	0.85	0.448	15.3 ± 3.0
Leicestershire 2, UK	17.8 ± 2.16	22.1 ± 1.4	1.24	0.169	27.6 ± 5.3
Derbyshire, UK	11.8 ± 0.57	9.5 ± 1.49	0.81	0.228	26.4 ± 4.5
Lithuania 1	18.4 ± 1.30	22.1 ± 0.24	1.20	0.049	30.9 ± 2.8
Lithuania 2	16.1 ± 0.93	25.0 ± 1.0	1.56	0.003	23.4 ± 1.4
Suffolk 1, UK	6.82 ± 0.87	4.5 ± 0.23	0.66	0.058	5.12 ± 0.68
Suffolk 3, UK	4.37 ± 0.18	2.9 ± 0.15	0.66	0.003	3.26 ± 1.03
Bulgaria 2	5.80 ± 0.36	4.1 ± 0.41	0.71	0.036	2.06 ± 1.12
Bulgaria 3	6.28 ± 0.69	5.3 ± 0.14	0.84	0.249	1.96 ± 1.01
Cambridgeshire 1, UK	9.79 ± 0.37	8.2 ± 0.32	0.84	0.034	5.65 ± 1.95
Norfolk 2, UK	6.18 ± 0.27	4.2 ± 0.41	0.68	0.015	2.13 ± 0.38
Northamptonshire 2, UK	5.24 ± 0.42	3.4 ± 0.39	0.65	0.030	4.83 ± 1.67
Cambridgeshire 2, UK	12.7 ± 0.09	10.3 ± 1.22	0.81	0.116	7.78 ± 2.15
North Yorkshire 2, UK	18.5 ± 0.48	14.9 ± 0.31	0.81	0.003	16.5 ± 3.1
Northern Ireland, UK	6.30 ± 0.33	6.7 ± 0.34	1.06	0.476	15.2 ± 4.2
North Yorkshire 3, UK	6.97 ± 0.60	5.4 ± 0.72	0.77	0.174	4.90 ± 1.53
North Yorkshire 4, UK	6.79 ± 0.45	4.7 ± 0.31	0.69	0.019	4.99 ± 2.06
North Yorkshire 5, UK	10.0 ± 0.06	9.0 ± 1.3	0.90	0.477	7.41 ± 1.25
North Yorkshire 6, UK	8.75 ± 0.34	7.3 ± 0.41	0.83	0.055	13.6 ± 3.1
Essex 3, UK	6.86 ± 0.71	5.4 ± 0.18	0.79	0.122	35.7 ± 6.5
Berkshire, UK	6.23 ± 0.12	4.2 ± 0.30	0.67	0.003	4.07 ± 1.10
Midlands, UK	5.28 ± 0.51	4.7 ± 0.31	0.89	0.395	6.12 ± 1.82
Devon 2, UK	8.68 ± 0.43	5.6 ± 0.23	0.65	0.003	7.52 ± 1.62
Buckinghamshire, UK	17.4 ± 0.96	13.1 ± 1.5	0.75	0.071	28.4 ± 6.0
Norfolk 3, UK	3.67 ± 0.30	2.5 ± 0.14	0.68	0.028	3.47 ± 0.92
Norfolk 4, UK	4.19 ± 0.21	2.9 ± 0.04	0.69	0.004	3.60 ± 0.99
Pentamidine (µM)	0.0027 ± 3.90E-04	0.6 ± 0.01	222	<0.0001	N.D.
Diminazene (µM)	N.D.	N.D.			0.37 ± 0.12

Effective Concentration 50% (EC_50_) values (µg/ml) are given as averages and SEM of 3 independent experiments for *T. brucei* and 3–4 experiments for *T. congolense*. P value is based on a Student’s unpaired t-test, comparing *T. brucei* WT and B48. R. I. is the resistance index, being the ratio of the EC_50_ values for *T. brucei* WT and B48. N.D., not determined.

**Figure 4:**
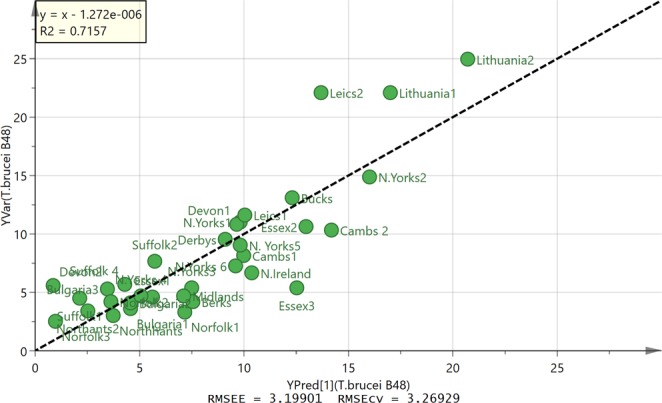
OPLS plot of observed against predicted activity against *T. brucei* B48 for 33 propolis samples based on five components.

**Figure 5:**
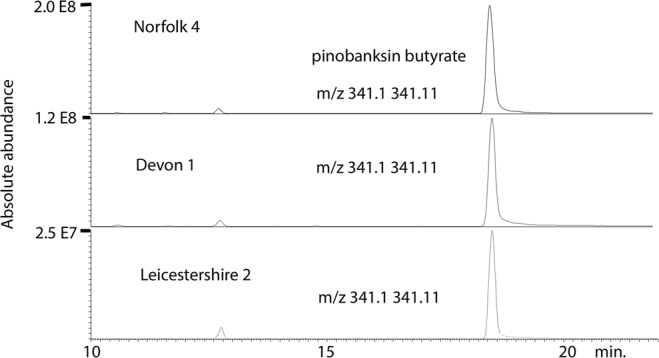
Extracted ion traces pinobanksin butyrate in samples with high, moderate and low activity against *T. brucei*.

OPLS was used to model the activity of the different propolis samples against *T. brucei* B48 in relation to their composition. It was possible to produce a model for 33 of the samples based on 5 components, including a butyl ester of pinobanksin, which produced a reasonable fit of predicted against observed activity shown in Fig. 4 (the corresponding loadings plot is shown in Fig. S1). The highest activity was associated with a butyl ester of pinobanksin and a propionyl ester of pinobanksin. Table S1 includes MS^n^ data used to further characterise the compounds associated with high activity. It can be seen from the extracted ion trace shown in Fig. 5 that the highest activity sample from Norfolk contains about 4 times the concentration of pinobanksin butyrate present in the lowest activity sample from Leicestershire. The wild type strain of *T. brucei* 427 gave similar results. Figure S2 shows an OPLS plot of predicted against measured activity with the corresponding loadings plot shown in Fig. S3. The highest activity is again associated with a butyl ester of pinobanksin and two propionyl esters of pinobanksin.

**Figure 6:**
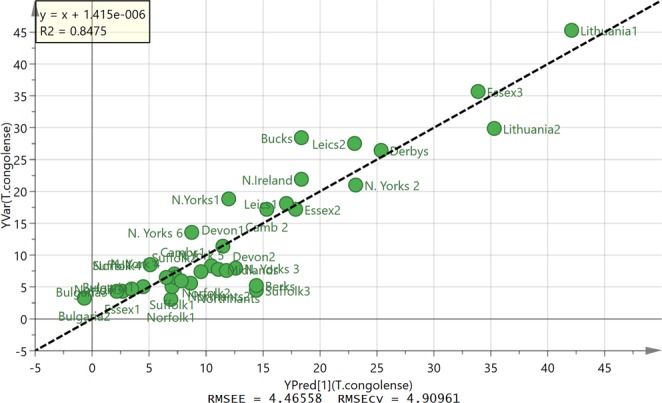
OPLS plot of observed against predicted activity against *T. congolense* for 35 propolis samples based on seven components.

The same propolis samples were also tested against the veterinary trypanosome species *T. congolense* (Table 1) with very similar results, as the average of the ratio of EC_50_ (Tbb427WT)/EC_50_(*T. congolense*) was 1.21 ± 0.11. Interestingly, the two Bulgarian samples were ~3-fold more active against *T. congolense* than against either of the *T. brucei* clones, as was one sample from Norfolk, UK. Figure 6 shows the OPLS plot obtained for the activity against *T. congolense*. The correlation between composition and activity was based on seven components. Figure S4 shows the corresponding loadings plot. There was a stronger fit for this plot than for the activity against *T. brucei* B48 and all 35 samples could be included in the model. Most active components against *T. congolense* are different from the most active against *T. brucei* and thus the OPLS plot highlights, galangin, an isomer of kaempferol, and a methylether of chrysin as the most active components (Table S1).

**Figure 7:**
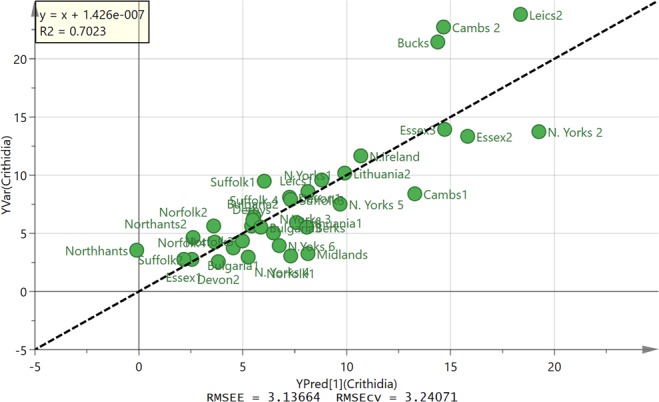
OPLS model of predicted against observed activity for propolis against *C. fasciculata* based on 4 components.

**Figure 8:**
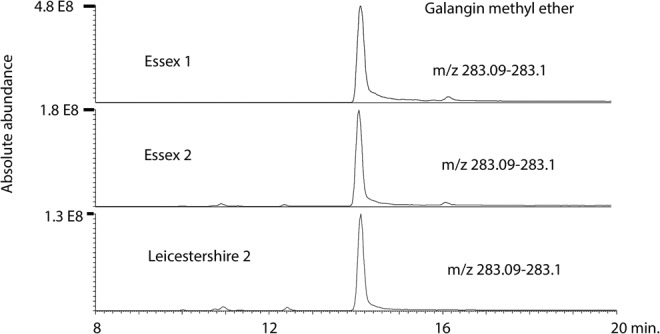
Extracted ion traces for galangin methyl ether in samples with high, moderate and low activity against *C. fasciculata*.

Table 2 shows the data obtained from testing propolis against *C. fasciculata* which is a closer relative to the trypanosomatids that infect bees than *T. brucei* is. A wide range of activities were obtained. In many cases the samples were less active against *C. fasciculata* than against *T. brucei*. The OPLS model did not give as strong a correlation with the components in the sample as for *T. congolense* (Fig. 7) although it was possible to reduce the number of variables supporting the plot to thus giving a better indication of which components might be associated with high activity. The corresponding loadings plot is shown in Fig. S5. Galangin methyl ether is associated with high activity and this can be seen in Fig. 8 where one of the most active samples from Essex has about four times the amount of this component in comparison to a sample from Leicestershire.

**Table 2:** EC_50_ values (µg/ml) for European propolis against *C. fasciculata* (n = 3).

Propolis	*C. fasciculate* EC_50_ AVG ± SEM	Ratio EC_50_(Tbb)/EC_50_(Cf)	P value
Suffolk 4, UK	6.41 ± 0.22	1.16	0.0798
Bulgaria 1	3.78 ± 0.65	1.37	0.1048
Suffolk 2, UK	2.80 ± 0.47	2.39	0.0029
North Yorkshire 1, UK	8.56 ± 1.19	1.57	0.0215
Northamptonshire 1, UK	3.54 ± 0.20	1.27	0.0324
Essex 1, UK	2.72 ± 0.23	2.20	0.0004
Essex 2, UK	13.4 ± 0.94	1.05	0.5182
Norfolk 1, UK	3.05 ± 0.48	1.71	0.0340
Devon 1, UK	8.11 ± 1.43	1.06	0.7664
Leicestershire 1, UK	9.58 ± 0.25	1.43	0.0269
Leicestershire 2, UK	23.8 ± 1.85	0.75	0.1030
Derbyshire, UK	5.64 ± 0.68	2.09	0.0022
Lithuania 1	5.92 ± 0.03	3.10	0.0007
Lithuania 2	10.1 ± 1.56	1.59	0.0310
Suffolk 1, UK	9.46 ± 1.03	0.72	0.1213
Suffolk 3, UK	7.94 ± 0.70	0.55	0.0077
Bulgaria 2	6.11 ± 0.66	0.95	0.6931
Bulgaria 3	5.55 ± 0.57	1.13	0.4633
Cambridgeshire 1, UK	8.44 ± 0.69	1.16	0.1597
Norfolk 2, UK	5.64 ± 0.93	1.10	0.6068
Northamptonshire 2, UK	4.62 ± 0.56	1.13	0.4258
Cambridgeshire 2, UK	22.7 ± 1.06	0.56	0.0007
North Yorkshire 2, UK	13.7 ± 1.15	1.35	0.0187
Northern Ireland, UK	11.6 ± 0.77	0.54	0.0032
North Yorkshire 3, UK	5.04 ± 0.71	1.38	0.1062
North Yorkshire 4, UK	2.95 ± 0.25	2.30	0.0018
North Yorkshire 5, UK	7.46 ± 1.00	1.34	0.0647
North Yorkshire 6, UK	3.98 ± 0.15	2.20	0.0002
Essex 3, UK	14.0 ± 0.99	0.49	0.0043
Berkshire, UK	5.56 ± 0.70	1.12	0.4015
Midlands, UK	3.27 ± 0.54	1.62	0.0540
Devon 2, UK	2.58 ± 0.43	3.36	0.0006
Buckinghamshire, UK	21.4 ± 1.34	0.81	0.0716
Norfolk 3, UK	4.34 ± 0.35	0.84	0.2208
Norfolk 4, UK	4.21 ± 0.49	1.00	0.9715
PAO^a^ (µM)	5.35 ± 4.72	5.44	5.17

^a^PAO = phenylarsine oxide.

**Figure 9:**
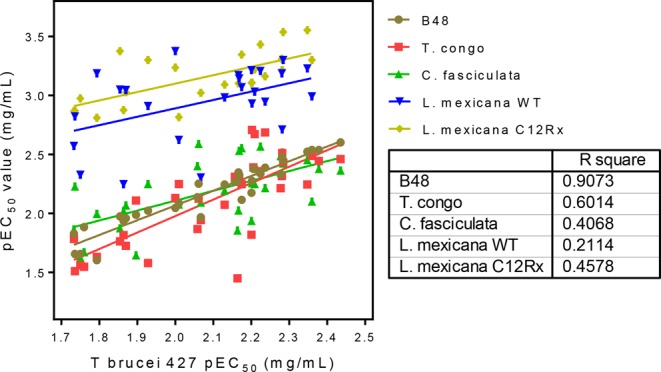
Correlation between the EC_50_ values of propolis samples against *T. brucei* 427WT and the other parasite strains and species.

Table 3 shows the activity obtained for 25 of the propolis samples against *L. mexicana*. The activity of the propolis samples against *L. mexicana* was higher than that obtained against *T. brucei*, with average EC_50_ values below 1 µg/mL for 52% of samples, and all EC_50_ values were under 5 µg/mL. The highest activity was obtained for one of the Bulgarian samples, at 0.35 ± 0.03 µg/mL. In most cases activity was equal or superior against the miltefosine APC12-resistant cell line, giving an average Resistance Index of 0.74 ± 0.09, but it was not possible to fit a strong an OPLS model for the data obtained for *L. mexicana* as for the *T. brucei* data, probably because the range of activities obtained across the samples is lower than for *T. brucei* and the number of samples tested was smaller. The activities obtained against *Leishmania* were an order of magnitude higher than those obtained for *C. fasciculata* and *T. brucei*, as shown in Fig. 9.

**Table 3:** The activity (µg/ml) of propolis against wild type and miltefosine-APC12 resistant *L. mexicana* (C12Rx).

Propolis ID	*L. mexicana* wild type	*L. mexicana* C12Rx	Resistance Index	ttest
	(µg/mL)	(µg/mL)		
Suffolk 4, UK	1.04 ± 0.19	0.81 ± 0.15	0.78	0.40
Bulgaria 1	0.35 ± 0.03	0.29 ± 0.04	0.85	0.33
Suffolk 2, UK	0.85 ± 0.14	0.45 ± 0.03	0.53	0.048
North Yorkshire 1, UK	0.90 ± 0.17	0.94 ± 0.15	0.96	0.87
Northamptonshire 1, UK	0.59 ± 0.05	0.28 ± 0.08	0.48	0.029
Essex 1, UK	0.62 ± 0.07	0.37 ± 0.07	0.60	0.073
Essex 2, UK	0.89 ± 0.10	0.42 ± 0.09	0.47	0.027
Norfolk 1, UK	1.94 ± 0.44	0.61 ± 0.003	0.31	0.027
Devon 1, UK	4.97 ± 0.23	0.95 ± 0.16	0.25	0.00014
Leicestershire 1, UK	5.67 ± 0.43	1.33 ± 0.09	0.23	0.00058
Leicestershire 2, UK	4.71 ± 0.33	1.06 ± 0.02	0.23	0.00041
Derbyshire, UK	1.23 ± 0.08	0.50 ± 0.17	0.41	0.016
Lithuania 1	1.51 ± 0.06	1.35 ± 0.02	0.89	0.064
Lithuania 2	0.65 ± 0.12	1.55 ± 0.01	2.38	0.0018
Suffolk 1, UK	0.67 ± 0.05	0.79 ± 0.09	1.17	0.32
Suffolk 3 UK	1.02 ± 0.18	0.50 ± 0.04	0.49	0.048
Bulgaria 2	1.13 ± 0.17	0.69 ± 0.22	0.61	0.19
Bulgaria 3	1.17 ± 0.18	0.78 ± 0.11	0.67	0.14
Cambridgeshire 1, UK	2.38 ± 0.40	1.53 ± 0.21	0.64	0.13
Norfolk 2, UK	0.93 ± 0.06	0.60 ± 0.05	0.65	0.020
Northamptonshire 2, UK	0.65 ± 0.05	0.49 ± 0.002	0.78	0.018
North Yorkshire 2	2.68 ± 0.15	1.36 ± 0.08	0.51	0.003
Northern Ireland	0.61 ± 0.05	0.78 ± 0.17	1.27	0.17
North Yorkshire 4, UK	0.72 ± 0.22	0.67 ± 0.06	0.94	0.75
North Yorkshire 5, UK	0.42 ± 0.12	0.58 ± 0.07	1.38	0.12
Miltefosine APC 12	0.1 ± 0.03	67.0 ± 12.6	670	<0.001
Miltefosine APC 16	2.0 ± 0.20	56 ± 9.7	28	<0.001

All EC_50_ values are given as average ± SEM (n = 3). Statistical difference between EC_50_ values of the same sample against two strains was analysed using Student’s unpaired t-test.

## Discussion

The importance of propolis to bees is not entirely clear, in so far as some strains of bee do not collect much of it. However, experimental work has been carried out in order to establish the role of propolis in protecting the hive against infection^21–29^. There is evidence that bees that collect greater amounts of propolis are healthier and produce more viable broods than bees which are selected for reduced propolis collection^21^. Bees that collected propolis were found to exhibit superior hygienic behaviour in comparison with those that collected less^22^. It was found that a parasite challenge encouraged bees to collect more propolis and that the propolis envelop improved the immunity of colonies against infection^23–27^. As in the current study, regional variations in the antimicrobial properties of propolis have been found to exist^28^. Several acyl esters of flavonoids were recently isolated from temperate propolis and were fully characterised by spectroscopic methods. The isolated compounds were tested against honey bee pathogens *Paenibacillus larvae* (American foulbrood) and *Ascosphaera apis* (chalkbrood). The most active compound against *A. apis* was pinobanksin 3-butyrate while the most active compound against *Paenibacillus larvae* was pinobanksin 3-octanoate^29^. The OPLS model for activity against *T. brucei* reflects this with two butyrate esters of pinobanksin being associated with the highest activity samples. In the current case the EC_50_ values against *T. brucei* in µg/ml terms are similar to those obtained for purified pinobanksin butyrate tested against *A. apis*. Interestingly the most active acyl flavonoid tested against *Paenibacillus larvae* was different from the most active against *A. apis* and this would seem to be the same in the current case, particularly for *C. fasciculata*, where the most active components were a methyl ether of galangin, a methyl ether of pinobanksin and pinobanksin. Thus propolis would appear to have broad spectrum activity with individual components in the mixture having activity against different organisms. Figure 9 shows a good overall correlation between the effects of the various samples against each of the kinetoplastid species. Especially between *T. brucei* and *T. congolense* the correlation is very close, which is important as African animal trypanosomiasis is caused by multiple *Trypanosoma* species including *T. congolense*, *T. b. brucei* and, in Eastern Africa, *T. b. rhodesiense*^30^ and the disease has now spread far beyond Africa for *T. vivax* and *T. evansi*^31^. Even more important is that the correlation between the drug-resistant and the sensitive strains was very good, with activity against the resistant strains on average better than against the parental strains. This is in line with previous observations that cross-resistance with current drugs will not be a problem for propolis-derived phytochemicals^3,6^, although it cannot be denied that resistance to any new, propolis-derived compound is likely to arise at some point unless a suitable combination therapy can be devised^32^.

The consistent high levels of activity obtained for propolis extracts against protozoa coupled with the prevalence of protozoal DNA amongst the DNA of pathogenic species in the metagenome^16^ of the bee suggests that these organisms may exert a greater pressure than may be currently appreciated on bee health. There remains much to understand about the role of propolis in bee health and also with regard to its potential in treating human infections, and the broad anti-kinetoplastid activity of propolis components reported here gives ample scope for further investigations.

## Materials and Methods

### Chemicals and materials

Absolute ethanol, HPLC grade acetonitrile, methanol, formic acid, water and Acrodisc syringe filters were obtained from Fisher Scientific (Loughborough, UK). 36 raw propolis samples were collected from different areas of the UK and Europe following a request by Mr James Fearnley for people to submit samples for testing. Miltefosine analogues APC12 and APC16 were obtained from Anatrace (Ohio, USA).

### Extraction of propolis samples

A sample of each propolis sample (500 mg) was extracted with 10 ml of ethanol by sonication for 1 h. The solvent was evaporated under a stream of nitrogen and the extracts were weighed and then redissolved in 5 ml of ethanol and then aliquoted into volumes containing 10 mg which were then blown to dryness under a stream of nitrogen.

### LC-MS Conditions

LC-MS was carried out by using an Accela pump connected to an Orbitrap Exactive mass spectrometer operated in positive/negative switching mode. The sheath gas and auxiliary gas were set at 50 and 17 arbitrary units, respectively. The needle voltage was 4.5 kV in positive mode and 4.0 kV in negative mode. The heated capillary temperature was 320 °C. The HPLC was fitted with an ACE C18 column 150 × 4.6 mm, 3 µM particle size (Hichrom, Reading, UK). Solvent A was 0.1% formic acid in water and solvent B was 0.1% formic acid in acetonitrile. The flow rate was 0.3 ml/min and the solvent gradient was as follows: 0 min 30% B, 30 min 100% B, 40 min 100% B, 41 min 30% B, 50 min 30% B. The files were processed by using m/z Mine 20.1 and then the masses were searched against an in-house database. The extracted data was then processed by using Simca P 14.1 (Umetrics, Umea, Sweden). To produce PCA and OPLS models^33,34^. MS^n^ experiments for characterisation of the activity marker compounds were carried out on an LTQ Orbitrap with a collision energy of 35 V and used the chromatographic and mass spectrometry conditions given above.

### Strains and cultures

Bloodstream forms of *T. b. brucei* were grown in standard HMI-9 medium with 10% fetal bovine serum at 37 °C/5% CO_2_, in vented culture flasks, exactly as described)^35^. The standard laboratory strain Lister 427WT^36^ was used as drug sensitive standard and the multi-drug resistant clone B48^37^ was used to assess the potential for cross-resistance with the diamidine and melaminophenyl arsenical classes of trypanocides. *T. congolense* strain IL3000 (Savannah-type) was cultured as described previously in Minimal Essential Medium (MEM) base with 10% goat serum, supplemented with 14 µL/L β-mercapto-ethanol, glutamine and antibiotics as described^38^.

Transgenic *Leishmania mexicana* promastigotes (5 × 10^6^ cells.ml) of strain MYNC/BZ/62/M379 expressing the firefly luciferase gene and sensitive to the miltefosine APC12 with 12 alkyl carbon chain called APC12^39^ were designated WT; a related strain, C12Rx, resistant to 80 µg/mL APC12, was selected under controlled conditions by a stepwise progressive increase of APC12 (Fig. S6), with surviving stationary phase cells at each dose, used to inoculate subsequent cultures. Cells able to grow in the presence of the drug were cloned under drug pressure by limiting dilution to 1 cell/ml in 20 ml of growth medium and plated out into 96-well plates. Both were cultured in complete Modified Eagle’s Medium (M199 supplemented with 10% (v/v) heat inactivated foetal calf serum) at 25 °C. The transgenic line cultures were further supplemented with Hygromycin B in order to retain the luciferase gene.

A standard wild-type *C. fasciculata* (strain HS6, kind gift of Professor Terry K. Smith, University of St-Andrews, UK) was grown at 27 °C in axenic serum-free defined media containing yeast extract (5 mg/mL), tryptone (4 mg/mL), sucrose (15 mg/mL), triethanolamine (4.4 mg/mL) and Tween 80 (0.5%) and supplemented with 10 μg/mL of haemin, exactly as described by Kipandula *et al*.^40^.

### Testing against *T. brucei*, *T. congolense* and *C. fasciculata*

The extracts were tested against *T. brucei* as described previously^3,6^, using our standard Alamar blue^®^ (resazurin) method in white opaque 96 well plates (Greiner Bio-One, Frickenhausen, Germany), with 23 doubling dilutions and a no-drug control for each sample, using 2 × 10^4^ *T. brucei* or 5 × 10^4^ *T. congolense* per well and incubating 48 h with test compound prior to the addition of resazurin sodium salt (Sigma) and a further incubation of 24 h. The method is based on live but not cells metabolizing blue, non-fluorescent resazurin to pink, fluorescent resorufin, with fluorescence intensity being proportional to cell numbers^41^. Stock solutions of each compound or mixture prepared in DMSO for each concentration so that there was a constant percentage of DMSO per well (1% v/v).

Testing against *C. fasciculata* involved a very similar procedure, using 5 × 10^3^ cells/well and incubations of 48 h and 24 h (27 °C, 5% CO_2_) before and after the addition of resazurin, respectively. Cell densities were determined using a haemocytometer after adding 1% v/v glycerol to the culture sample to immobilize the parasites. Cell density was then adjusted to 5 × 10^4^ cells/mL with fresh medium, of which 100 µL was added to each well of a pre-prepared 96-well plate with the doubling dilution of test compound/sample.

Fluorescence was determined using a FLUOstar Optima (BMG Labtech, Durham, NC, USA) plate reader (λ_ex_ = 544 nm; λ_em_ = 590 nm) and the output was plotted to a sigmoid curve with variable slope (Prism 5.0, GraphPad software) to obtain 50% effective concentrations (EC_50_ values).

### Testing against *L. mexicana*

A miltefosine APC12-resistant *L. mexicana* was strain was selected as shown in Fig. S6. Both cell lines were screened with propolis samples at a starting concentration of 0.125 mg/ml, doubly diluted eleven times across a 96 well plate in triplicate and incubated for 72 h at 25 °C. Wells with no propolis added were used in control experiments. After, luciferin solution (1 µg/ml) was added and the light emitted was measured using a luminometer (Biotek Synergy HT) at a wavelength of 440/40 nm. Viability was taken to be proportional to light emitted from for each drug-treated well, and was expressed as a faction of emission from the ‘no drug’ control. IC_50_ values were determined using Prism 5.0, GraphPad software.

## Supplementary information

Supplementary Information

## Data Availability

The datasets generated during and/or analysed during the current study are available from the corresponding author on reasonable request.
