# Supplementary material for: European propolis is highly active against trypanosomatids including Crithidia fasciculata
Source: Sci Rep. 2019 Aug 6;9:11364. doi: 10.1038/s41598-019-47840-y (PMC6684629; doi:10.1038/s41598-019-47840-y)
Supplement: Supplementary file 1 — Supplementary Information [file 41598_2019_47840_MOESM1_ESM.docx]

**European propolis is highly active against trypanosomatids including *Crithidia fasciculata***

Abdullah Alotaibi^1^, Godwin U. Ebiloma^2^, Roderick Williams^3^, Sameah Alenezi^1^, Anne-Marie Donachie^2^, Selome Guillaume^3^, John O. Igoli^1,4^, James Fearnley^5^, Harry P. de Koning^2^ and David G. Watson^1*^.

1. University of Strathclyde, Strathclyde Institute of Pharmacy and Biomedical Science, 161 Cathedral Street, Glasgow, G4 0RE, UK.
2. Institute of Infection, Immunity and Inflammation, College of Medical, Veterinary and Life Sciences, University of Glasgow, Glasgow G12 8TA, UK
3. IBEHR, School of Health and Life Science, University of the West of Scotland, High Street, Paisley PA1 2BE
4. Department of Chemistry, University of Agriculture, PMB 2373, Makurdi, Nigeria
5. BeeVital, Whitby, North Yorkshire, YO22 5JR, UK.

*Corresponding author

[d.g.watson@strath.ac.uk](mailto:d.g.watson@strath.ac.uk)

+441415482651

**Supplementary Information**

**Figure S1** Loadings plot corresponding to figure 4 for an OPLS model of propolis activity against *T.brucei* B48.


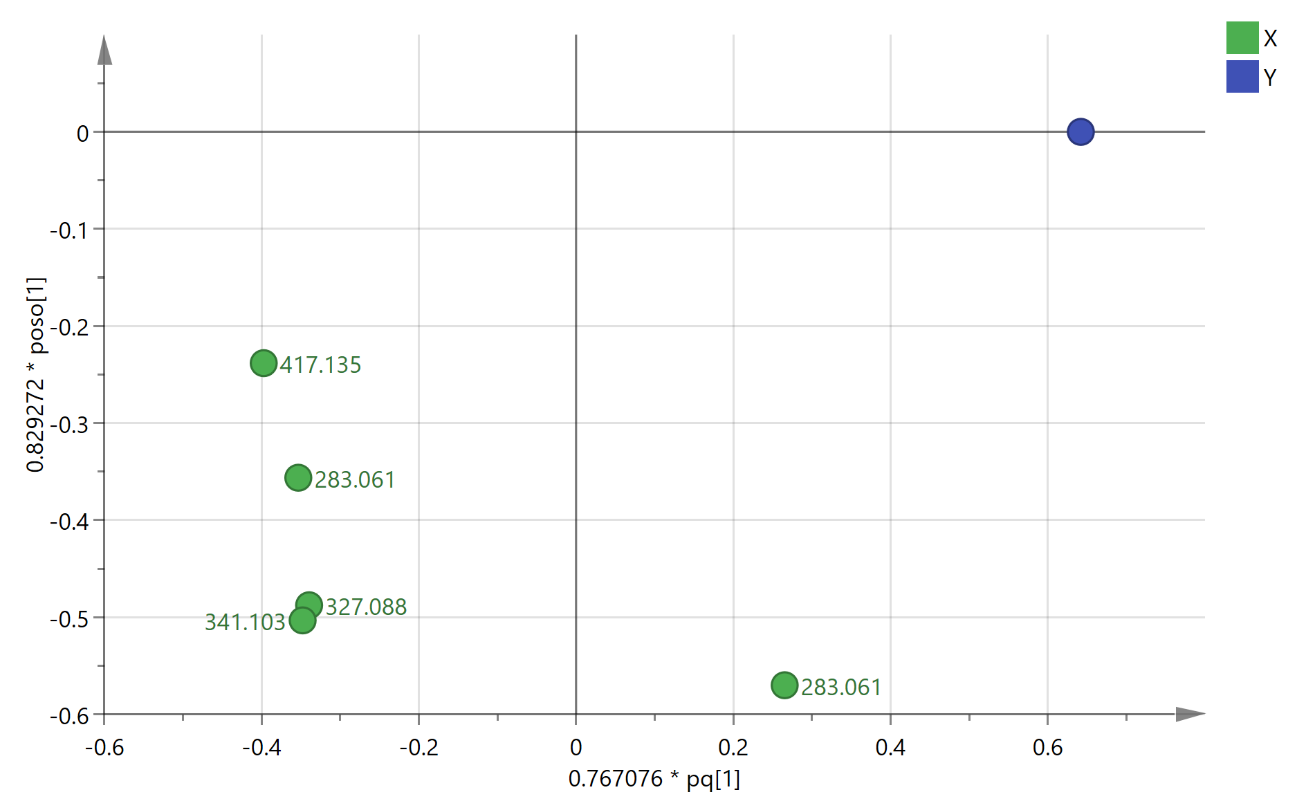


**Figure S2** OPLS model for observed against predicted activity of 33 propolis against *T. brucei* 427 WT based on 7 components.


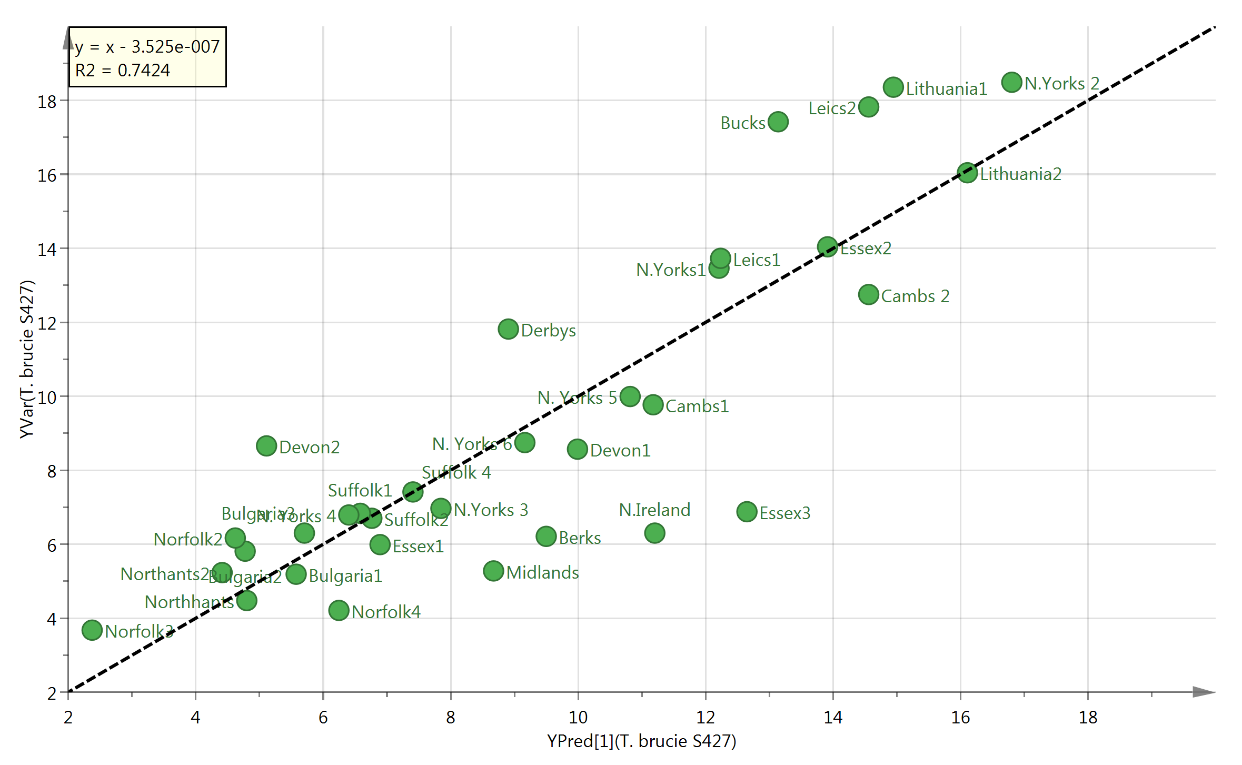


**Figure S3** The loadings plot corresponding to figure S2 which shows a plot of predicted against observed activity for 33 propolis samples against *T. brucei* 247 WT.


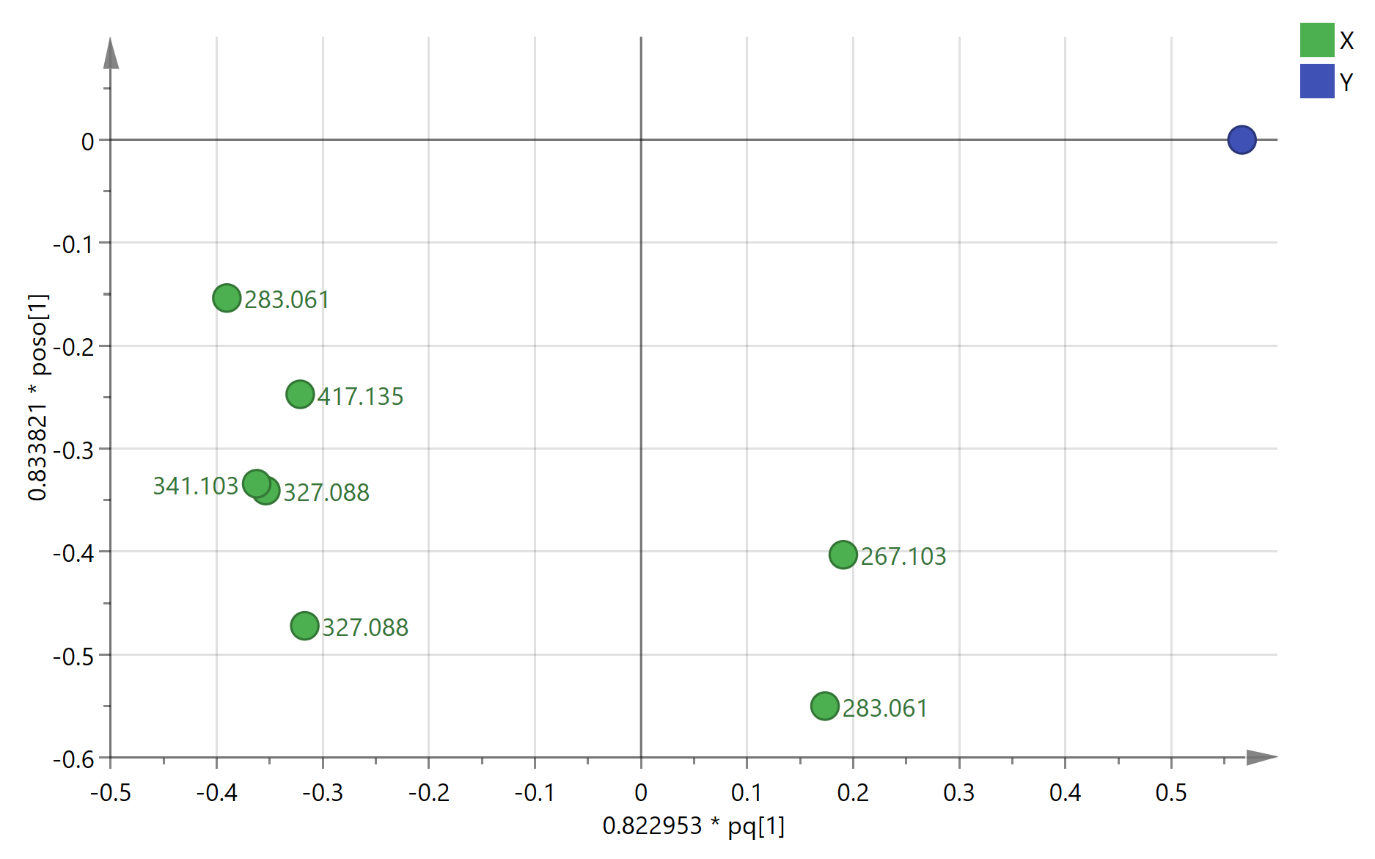


**Figure S4** The loadings plot corresponding to figure 6 which shows a plot of predicted against observed activity of 35 propolis samples against *T. congolense*.


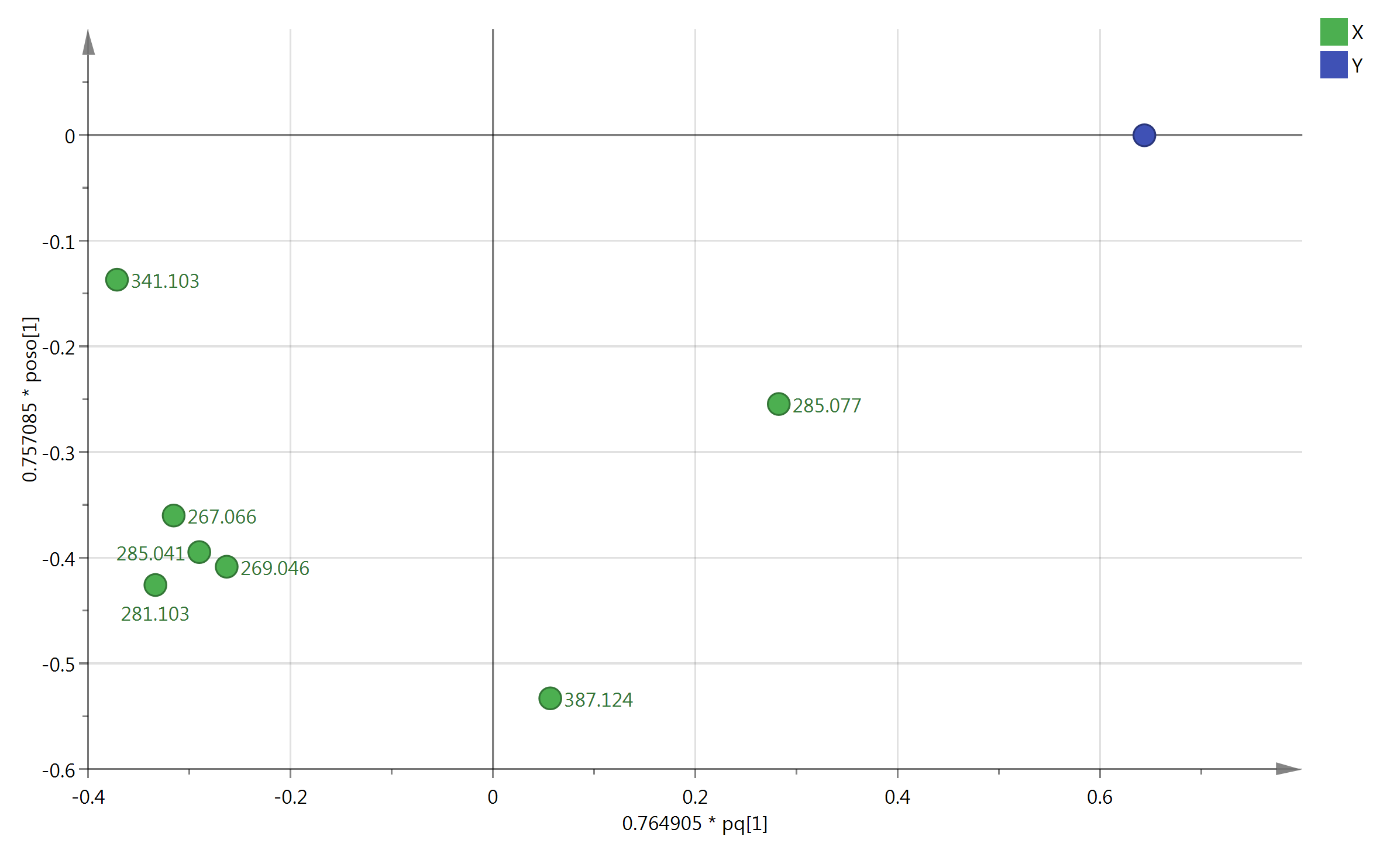


**Figure S5** Loadings plot corresponding to figure 7 which shows a plot of predicted against observed activity of 35 propolis samples against *C. fasciculata*.


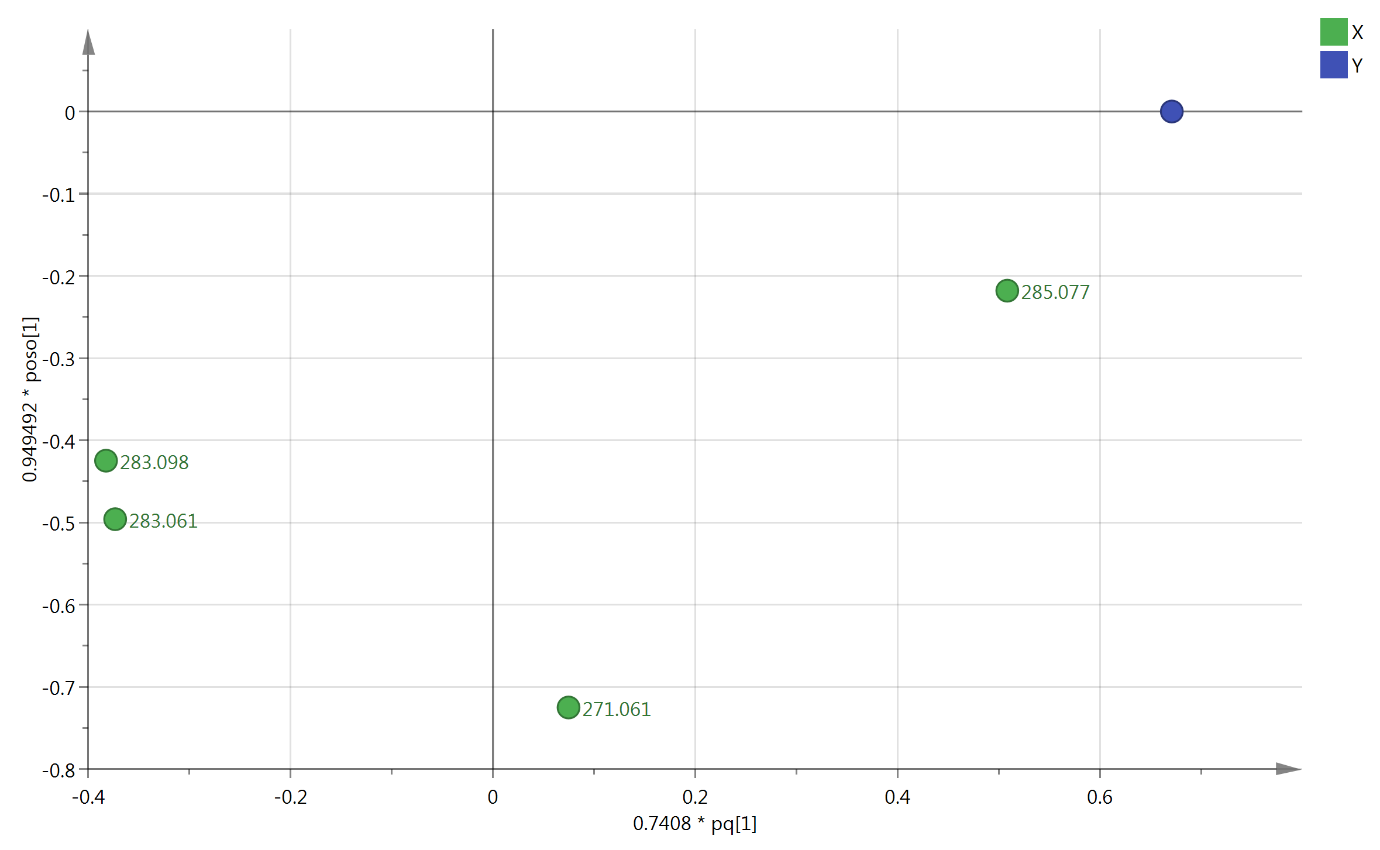


**Figures S6** Selection of APC12-resistance in *L. mexicana* promastigotes.

*L. mexicana* promastigotes designated C12Rx were selected under controlled conditions over 8 passages (150 days) in increasing concentrations of miltefosine analogue APC12. Adaptation continued until the population was resistant to 80 µg/ml, APC12 in the medium, beyond which death ensued. The IC_50_ of C12Rx was 67.0 µg/ml and 56.0 µg/ml for APC12 and APC16 (miltefosine) while for *L. mexicana* WT to the same drugs, they were 0.1 µg/ml and 2.0 µg/ml (resistance index 670 and 28 for APC12 and APC16 respectively).

**Table S1** MS^n^ data for the active compounds observed in OPLS plots of predicted and observed activity obtained with a collision energy of 35V.

| **Tentative id m/z** |  | **Rt min** | **[M-H]^-^ Composition** | **Fragments (relative intensity, elemental composition)** |
| --- | --- | --- | --- | --- |
| ***T. brucei* B48** | | | | |
| Dimethyl kaempferol phenethyl ether | 417.135 | 17.4 | C_25_H_21_O_6_ | 402.1098(95, C_24_H_18_O_6_), 387.086 (15, C_23_H_15_O_6_) 375.123 (3 C_23_H_19_O_5_) 298.0478 (100, C_16_H_10_O_6_)  MS^3^ (298.0478) 270.053(100, C_15_H_10_O_5_) |
| Galangin methyl ether | 283.061 | 15.4 | C_16_H_11_O_5_ | 268.038 (100, C_15_H_8_O_5_), 255.030 (65, C_14_H_7_O_5_)  MS^3^ (268.038) 239.035 (100, C_14_H_7_O_4_), 213.0399 (15.3, C_13_H_7_O_3_) |
| Galangin methyl ether | 283.061 | 14.4 | C_16_H_11_O_5_ | 268.038 (100, C_15_H_8_O_5_) |
| Pinobanksin butyrate | 341.103 | 18.5 | C_19_H_17_O_6_ | MS^2^ 253.050 (100, C_15_H_9_O_4_)  MS^3^ (253.050) 209.0605 (100, C_14_H_9_O_2_) 181.0657 (6.8, C_13_H_9_O) |
| Pinobanksin propionate | 327.088 | 16.5 | C_18_H_15_O_6_ | MS^2^ 253.051 (100, C_15_H_9_O_4_) |
| Pinobanskin propionate | 327.088 | 10.7 | C_18_H_15_O_6_ | MS^2^ 253.051 (100, C_15_H_9_O_4_) |
| ***T. brucei* 247 WT** | | | | |
| Pinobanksin butyrate | 341.103 | 18.5 | C_19_H_17_O_6_ | MS^2^ 253.050 (100, C_15_H_9_O_4_)  MS^3^ (253.050) 209.0605 (100, C_14_H_9_O_2_) 181.0657 (6.8, C_13_H_9_O) |
| Methyl ether of dihydrokaempferol | 301.072 | 9.3 | C_16_H_13_O_6_ | 273.077 (100, C_15_H_13_O_5_) 257.046 (6.7, C_14_H_9_O_5_) |
| Coumaric acid  phenyl propenyl ester | 279.103 | 18.5 | C_18_H_15_O_3_ | 235.1123 (100, C_17_H_15_O), 194.0814 (95, C_14_H_11_O) 162.0319 (15.8, C_9_H_6_O_3_) |
| Pinobanksin methyl ether | 285.077 | 15.5 | C_16_H_13_O_5_ | 270.053 (100, C_15_H_10_O_5_), 243.066 (100, C_14_H_11_O_4_)  164.011 (42, C_8_H_4_O_4_) |
| ***C. fasciculata*** | | | | |
| Pinobanksin methyl ether | 285.077 | 15.5 | C_16_H_13_O_5_ | 270.053 (100, C_15_H_10_O_5_), 243.066 (100, C_14_H_11_O_4_)  164.011 (42, C_8_H_4_O_4_) |
| Galangin methyl ether | 283.061 | 15.4 | C_16_H_11_O_5_ | 268.038 (100, C_15_H_8_O_5_), 255.030 (65, C_14_H_7_O_5_)  MS^3^ (268.038) 239.035 (100, C_14_H_7_O_4_), 213.0399 (15, C_13_H_7_O_3_) |
| Octyl ester of caffeic acid | 283.098 | 17.1 | C_17_H_15_O_4_ | 179.035 (100, C_9_H_7_O_4_) 135.045 (20, C_8_H_7_O_2_) |
| Pinobanksin | 271.061 | 9.3 | C_15_H_11_O_5_ | 253.050 (100, C_15_H_9_O_4_) 225.055 (21, C_14_H_9_O_3_) 197.061 (16, C_13_H_9_O_2_) 151.004(6.4, C_7_H_3_O_4_) |
| ***T. congolense*** | | | | |
| Pentenoyl ester of caffeic acid | 281.103 | 3.4 | C_14_H_17_O_6_ | 179.035 (78, C_9_H_7_O_4_) 135.045 (100, C_8_H_7_O_2_) |
| ‡Kaempferol isomer | 285.041 | 6.5 | C_15_H_9_O_6_ | 241.0504 (100, C_14_H_9_O_4_) 151.0036 ( 12.1%, C_7_H_3_O_4_) |
| Methyl ether of chrysin | 267.066 | 7.9 | C_16_H_11_O_4_ | 252.042 (100, C_15_H_8_O_4_), 224.047 (C_14_H_8_O_3_) 14.2 |
| Pinobanksin methyl ether | 285.077 | 15.5 | C_16_H_13_O_5_ | 270.053 (100, C_15_H_10_O_5_), 243.066 (100, C_14_H_11_O_4_)  164.011 (42, C_8_H_4_O_4_) |
| *Galangin | 269.046 | 8.8 | C_15_H_9_O_5_ | MS^2^ 225.056 (100, C_14_H_9_O_3_) 201.056 (23 C_12_H_9_O_3_) 149.024 (26, C_8_H_5_0_3_) |
| Unknown flavonoid | 387.124 | 18.4 | C_24_H_19_O_5_ | MS^2^ 359.129 (14.4, C_23_H_19_O_4_,), 343.134 (6.5, C_23_H_19_O_3_), 281.082 (49.3, C_17_H_13_O_4_), 267.066 (100, C_16_H_11_O_4_) |

*Retention time and fragmentation pattern corresponding to that of a standard. ‡ Elutes earlier than the standard.
